# Sustaining HIV Research in Resource-Limited Settings Using PLAN (People, Learning, Adapting, Nurturing): Evidence from the 4 Youth by Youth Project in Nigeria

**DOI:** 10.1007/s11904-023-00652-2

**Published:** 2023-03-29

**Authors:** Juliet Iwelunmor, Joseph D. Tucker, Oliver Ezechi, Ucheoma Nwaozuru, Chisom Obiezu-Umeh, Titilola Gbaja-Biamila, David Oladele, Adesola Z. Musa, Collins O. Airhihenbuwa

**Affiliations:** 1 Department of Behavioral Science and Health Education, College for Public Health & Social Justice, Saint Louis University, 3545 Lafayette Ave, Saint Louis, MO 63104 USA; 2 Department of Medicine, University of North Carolina at Chapel Hill, Chapel Hill, NC USA; 3 Faculty of Infectious and Tropical Diseases, London School of Hygiene and Tropical Medicine, London, UK; 4 Clinical Sciences Department, Nigerian Institute of Medical Research, Lagos, Nigeria; 5 Heath Policy and Behavioral Sciences, School of Public Health, Georgia State University, Atlanta, GA USA

**Keywords:** Sustainability, Implementation, Frameworks

## Abstract

**Purpose of Review:**

Sustaining evidence-based interventions in resource-limited settings is a perennial challenge. Despite growing research on the significance of sustainability, few frameworks describe why and how to plan for sustainability in settings limited with resources. Drawing on a synthesis of the literature on sustainability, including the Dynamic Sustainability Framework, we review lessons learned from research to date, to point out a path forward for sustaining evidence-based interventions in resource-limited settings.

**Recent Findings:**

We describe PLAN or *why people learning*, *adapting*, *and nurturing* the core values of an intervention can enhance its sustainability over time. PLAN is a dynamic framework that simplifies the process of planning for sustainability of evidence-based interventions throughout the lifecyle of an intervention, taking into consideration the people that matter as well as the learning, adaptation, and nurturing involved with understanding and studying the interactions between interventions/innovations, practice settings, intervention fit, and the broader ecological contexts in which implementation occurs. We use case-study data from our ongoing pragmatic HIV implementation trial, the 4 Youth by Youth project, to detail the value and implications of why people learning, adapting, and nurturing HIV interventions implemented in resource-limited settings matter.

**Summary:**

PLAN is designed to further the dialogue on ways research and practice teams can critically work to ensure the sustainability of their evidence-based interventions from the onset, particularly in settings and with populations limited with resources. It also illustrates how attention to sustainability from the beginning may foster actions necessary for sustained program → sustained benefits → sustained capacity → sustained value, but in the absence of early and active planning, none of this will occur. Ultimately, we hope to accelerate the sustainability of evidence-based HIV interventions, and making a *PLAN* at the bare minimum may ensure that the goals of continuing and maintaining desirable features of any evidence-based interventions can be realized.

## Introduction

What can be done from the onset or across the lifecycle of an intervention to sustain effective interventions and why does this matter? Many researchers have attempted to answer this fundamental question in implementation science [1–3]. We define sustainability here following, Scheirer and Dearing, “as the continued use of intervention components and activities for the continued achievement of desirable health outcomes within the population of interest [4].” We also note and according to Shediac-Rizkallah and Bone (1998) that it may include [1] when there is continuation of the core elements of at intervention; [2] when there is continuation of intended benefits (i.e., uptake of interventions); and [3] when adequate capacity for continuation of core elements is maintained [5].

Much of the literature on sustainability focuses on operational definitions, barriers, and facilitators [1–3, 6–15]. Moreover, published literature also offer 62 identified, distinct approaches necessary for sustainability [9], including recent reporting of evidence-based strategies that facilitate sustainability of interventions [12] and free-listing exercises on ways to conceptualize and measure sustainability [16]. Yet, few implementation trials (distinct from clinical trials) result in sustainable outcomes. In a systematic review of health interventions implemented in sub-Saharan Africa, less than half of the studies reviewed had any sustainability outcomes [1].

Similarly, and among researchers funded to conduct implementation research, only about two-thirds of the studies made references to sustainability, and none included any sustainability planning [2]. As a result, gaps remain between the outcomes of implementation trials and the sustainability of their outcomes within health and community settings [3, 14]. Planning, especially when made simple, and using theoretically informed approaches, may enhance and advance efforts to sustain evidence-based interventions (EBI).

This shift away from static to the dynamic conceptualization of sustainability is consistent with the Dynamic Sustainability Framework, which draws attention to the significance of planning. The goal is for “continuous learning and evaluation, problem-solving, improvement, and ongoing adaptation of evidence-based interventions to enhance fit with contexts and populations over time” [17]. Frameworks like Reach, Effectiveness, Adoption, Implementation, and Maintenance (RE-AIM) emphasize that the *reach*, *implementation*, *effectiveness*, or even *adoption* of any evidence-based intervention will not necessarily lead to maintenance without specific planning and action [18–21]. Yet, for all its familiarity, planning remains an underutilized critical step in strategies and efforts to sustain evidence-based interventions. Only recently has guidance on D&I process frameworks for early sustainability planning emerged, such as with the designing for dissemination and sustainability (D4DS) model that depicts a path from conceptualization to design to dissemination to impact [22]. However, D4DS does not account for multilevel determinants including socio-cultural and contextual factors that may potentially hinder or facilitate the ability to accomplish each phase. In the aforementioned study of how researchers conceptualized and planned for sustainability, few studies had a sustainability plan, thus lacking clarity around operationalizing sustainability [2]. Some used the weaning off approach whereby research personnel built and relied on the capacity of front-line implementers to assume the role of implementation leaders after the research study was completed or the research personnel left the setting [2]. Others utilized a strategic funding approach where researchers used research funds only for research activities, relying on securing buy-in from host organizations to fund implementation. Both strategies consider sustainability at the end and not the beginning of an intervention implementation. Too often, researchers fail to do enough, early enough to ensure sustainability, often leaving it as something to be dealt with in the later years once some results are in or when there is sufficient time after intervention implementation has begun [23]. Prior research suggests the time for doing something may never come, and if it does, it may be too late. As a result, expecting or hoping for sustainability, whether via weaning off or seeking strategic funding, never translates into doing something about it [23]. We are left still with limited understanding on why or how evidence-based interventions are sustained remains poorly understood, including the plans used to guide their sustainability.

The aim of this paper is to describe a sustainability strategy that focuses on PLAN, or *why people learning*, *adapting*, *and nurturing* the core values of an intervention can enhance its sustainability over time. The core values help to ensure that aspects of implementation trials are sustainable whether with [1] continuation of the core elements of at intervention; [2] continuation of intended benefits (i.e., uptake of interventions); and [3] adequate capacity for continuation of core elements is maintained [5]. PLAN is illustrated using a case study from the *4 Youth by Youth Project (4YBY)*, an ongoing implementation trial using innovative and pragmatic tools to expand youth-friendly HIV self-testing in Nigeria. We conclude the paper with a discussion on how public health agencies, funders, researchers, and intended beneficiaries can be thoughtfully engaged in planning the sustainability of HIV implementations. Engaging the key people to learn, adapt, and nurture their core values of any intervention is likely to ensure the continued use of intervention components and activities for the continued achievement of desirable outcomes overtime.

## Why PLAN?

We define PLAN here as how core values of evidence-based interventions with the key people can foster learning, adaptation, and nurturing of resources for continuous feedback and dialogue so that the intervention remains over time. PLAN originated from a prior systematic review we conducted where we argued for the need to understand not only what is to be sustained but the how-to-do-it literature on sustainability [1]. It also builds on the Dynamic Sustainability Framework (DSF), which emphasizes three things (i.e., the intervention, the practice setting or context, and ecological system) necessary for the sustainability of evidence-based interventions-context, ongoing evaluation, and continuous learning and improvement [17]. As a dynamic model, PLAN extends DSF’s emphasis on continuously optimizing the fit between the intervention and the context to achieve maximal benefit. Additional insights from recent studies and systematic reviews [2, 10, 12, 24, 25], for example, on ways to navigate the sustainability landscape [9], also guided the conceptualization of PLAN. Since sustainability is a dynamic process involving interactions between interventions/innovations, practice settings, intervention fit, and the broader ecological contexts [1, 17], a dynamic plan made simple from the onset is critical to ensure the continued use of intervention components overtime. Multiple principals of community-based participatory research (CPBR) also apply to PLAN, including [1] building upon the strengths and resources within the community; [2] facilitating collaborative, equitable partnership; [3] fostering co-learning and capacity building; [4] which involves a long-term process and commitment to sustainability [26].

## Components of PLAN

### Core Values

PLAN begins with identifying an evidence-based intervention’s core values. In the book “Built to Last,” Collins and Porras [27] defined values “as essential and enduring tenets, or timeless guiding principles, and operating practices and norms that never change.” As a powerful drive for action, values may play a central role in guiding and identifying key resources necessary for sustaining evidence-based interventions. Values help to clarify the overarching goals of intervention, serving as a rallying point not only for intervention implementers but also for key stakeholders themselves, whether at the individual, provider, health systems, community, or policy levels [28]. Value definitions are in alignment with our definition of sustainability and synthesis of the literature [29–31], and it may include a description of effect on patients as measured by change in morbidity, mortality, or quality of life compared with baseline or an alternative option as well as equity value, who benefits, who does not, and why. Value may also include an understanding from stakeholders themselves, such as what capacity is needed to continue aspects of the intervention. Time is also important in other to understand both upfront and long-term impact, as well as to compare change over time to existing solutions. Finally, value may be defined more broadly to include a dissemination value consideration, or how to maintain attention to the issues [30]. Additionally, and consistent with the Dynamic Sustainability Framework, values shape knowledge production for sustainability, ensuring that interventions are continually improved to boost sustainment in practice [17]. Values require ongoing stakeholder involvement with quality improvement where necessary to maximize the fit between an intervention and its delivery context and to improve the public health benefit of interventions [17]. Efforts to sustain evidence-based interventions should include an articulation of core values, to keep them, not only from the time they are identified or developed but throughout the life cycle of a project.

### People

Next, PLAN includes people. At the heart of any sustainability, the effort focuses on how people understand sustainability in practice [32]. Sustainability planning depends on assembling key group of stakeholders early in the process of intervention development and throughout the life-cycle of an intervention [32]. The key people may include program champions, long regarded as key facilitators for successful change efforts in health and community systems [33]. In alignment with the tenets of DSF, PLAN seeks to continuously engage key stakeholders to not only increase the fit between the intervention and the local context but also to build the buy-in necessary with relevant people, including the effective leadership, alignment of leadership, and organizational support across organizational levels, obtaining formal commitments and resource sharing agreements for the sustainability of evidence-based interventions [17, 34, 35]. As previous research suggests, better maintenance of research evidence requires collaborations between those involved in the process of generating research and those addressing the real-world needs and limitations of community or health [1] systems and their end-users [36]. Chambers and colleagues have suggested the importance of having input from end-users to ensure sustainability. Nonetheless, specific attributes of key people, including their influence, ownership of the interventions, physical presence in particular settings, persuasiveness, grit, and participative leadership may enhance sustainability of interventions [33].

### Learning

Learning here refers to collaborative and iterative problem-solving to consistently adhere to core values. Sustainability planning facilitates learning, particularly a constant vigilance of the core values identified. In its broadest sense, learning, especially in collaboratives or communities, is like the anthills of a savannah [37]. Even if the memories (of interventions) are short-lived, efforts to learn, and with the key people are the permanent structures that remain and live to tell the tale of the implementation [38]. Learning is a core component of DSF, given the constantly changing context in which interventions are implemented and sustained [17]. Learning helps to iteratively solve problems at multiple levels [17]. It may include, for example, learning communities working together to foster not only the identification of potential solutions, but also generate critical advice, apply quality improvement strategies, and exchange experiences and opportunities for enhancing the sustainability of evidence-based interventions [4, 39, 40]. Learning communities alter social networks among participants to promote the transmission of new ideas and the adoption or maintenance of existing values [41–43]. It creates a supportive infrastructure that can help sustain the delivery of an evidence-based intervention [44]. Learning also fosters ongoing dialogue, including what Airhihenbuwa describes as “polylogue” or joint co-creation of meanings [45], to determine how best to develop, deliver, and sustain evidence-based interventions. Taken together, learning here acknowledges that key stakeholders have a voice that should not be omitted or taken for granted as they play a crucial role in sustaining essential elements interventions that have a strong fit with particular contexts [1].

### Adapting

By adaptation, we refer to the thoughtful and deliberate alteration to the design or delivery of an intervention with the goal of improving its fit or effectiveness in a given context [46, 47]. Sustainability planning fosters an understanding of how evidence-based interventions’ values or core principles can be adapted within particular settings. Real-world settings are inherently messier and more unpredictable than the controlled settings used commonly in clinical trials [48, 49]. Adaptations, in turn, are like new grasses that grow or regenerate after the grasslands burn [38]. They are often improvements necessary to enhance the adoption and continuation of evidence-based interventions [50]. They may include changes to multiple features of an intervention to improve the fit between interventions and contexts [46, 47, 50]. Consistent with the Dynamic Sustainability Framework, adaptation includes a long-term recognition of an intervention’s need to evolve within and across contexts [50]. Also, with the Dynamic Adaptation Process [51], adaptation is the steps taken to allow an evidence-based practice to be delivered faithfully in situations where it otherwise might not fit. This includes identifying and distinguishing core elements and adaptable characteristics of an evidence-based intervention, whether at the client level, provider level, systems, or organizational levels [50]. It also echoes the tenets of FRAME-IS (Framework for Reporting Adaptations and Modifications Enhanced for Implementation Science) [46, 47, 50]. Specifically, if interventions are to be maintained, when and how modifications to evidence-based interventions occurred, whether planned or unplanned, their relationship to fidelity as well as the reasons and goals for modification should, in turn, be cataloged [46, 47].

### Nurturing

**Fig. 1:**
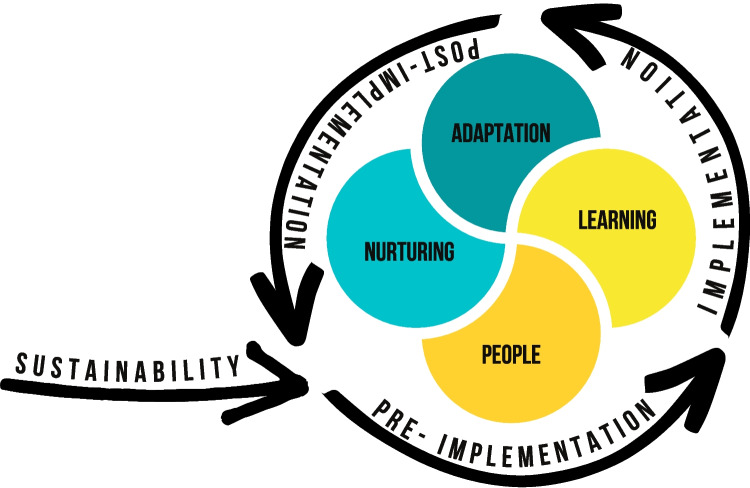
Overview of the PLAN approach

Finally, sustainability planning provides an opportunity for continuous feedback to nurture conditions and contexts, particularly those currently existing and likely to support the sustainability of effective evidence-based interventions. By nurturing and guided by the PEN-3 cultural model [52–54], we refer to supportive influences likely to contribute to the long-term maintenance of evidence-based practices within particular contexts. It includes supportive practices such as dialogues around what already exists within a given context. Dialogue allows all stakeholders to commit to contributing information and lessons that can support learning and decision-making. Following DSF, the dialogue around sustainability can foster the mobilization of existing resources around an intervention, help to weigh the benefits versus the risks, while revealing emerging opportunities and constraints arising within the intervention itself, within communities, and the broader ecological setting that may facilitate or hinder sustainability [1, 17, 54]. When maximized appropriately, these resources can go on to build more and bigger anthills or strategies that ensure the sustainability of the interventions or practice. One issue that appears significant in enhancing this process is trust. Working collaboratively to sustain evidence-based interventions will require not only gaining an understanding of the challenges facing both implementers and key stakeholders with delivering protocols within real-world settings but also careful and continuous attention to a range of activities, including dialogue that nurtures and builds an unwavering sense of trust and openness in collaborative relations [45, 55, 56]. This is because when both implementation scientists and key stakeholders come together, they bring different resources and expertise to the table, which in turn may enhance or limit efforts to sustain the evidence-based intervention in question. Yet, both researchers and implementers and key stakeholders often have different reasons for being involved, and sometimes, these reasons may not only lead to tensions, but also conflicts of interests and unclear goals, aims, or even values guiding how to sustain the evidence-based intervention. Nurturing relationships can build trust between partners [56]. Partners need to trust each other enough to form not only expectations but also the ability to weigh the benefits and risks with initiating and maintaining core values of interventions over time [56]. Figure 1 provides an overview of the PLAN approach.

## Application of PLAN: a Case Study Analysis from the 4Youth by Youth Project (4YBY)

**Fig. 2:**
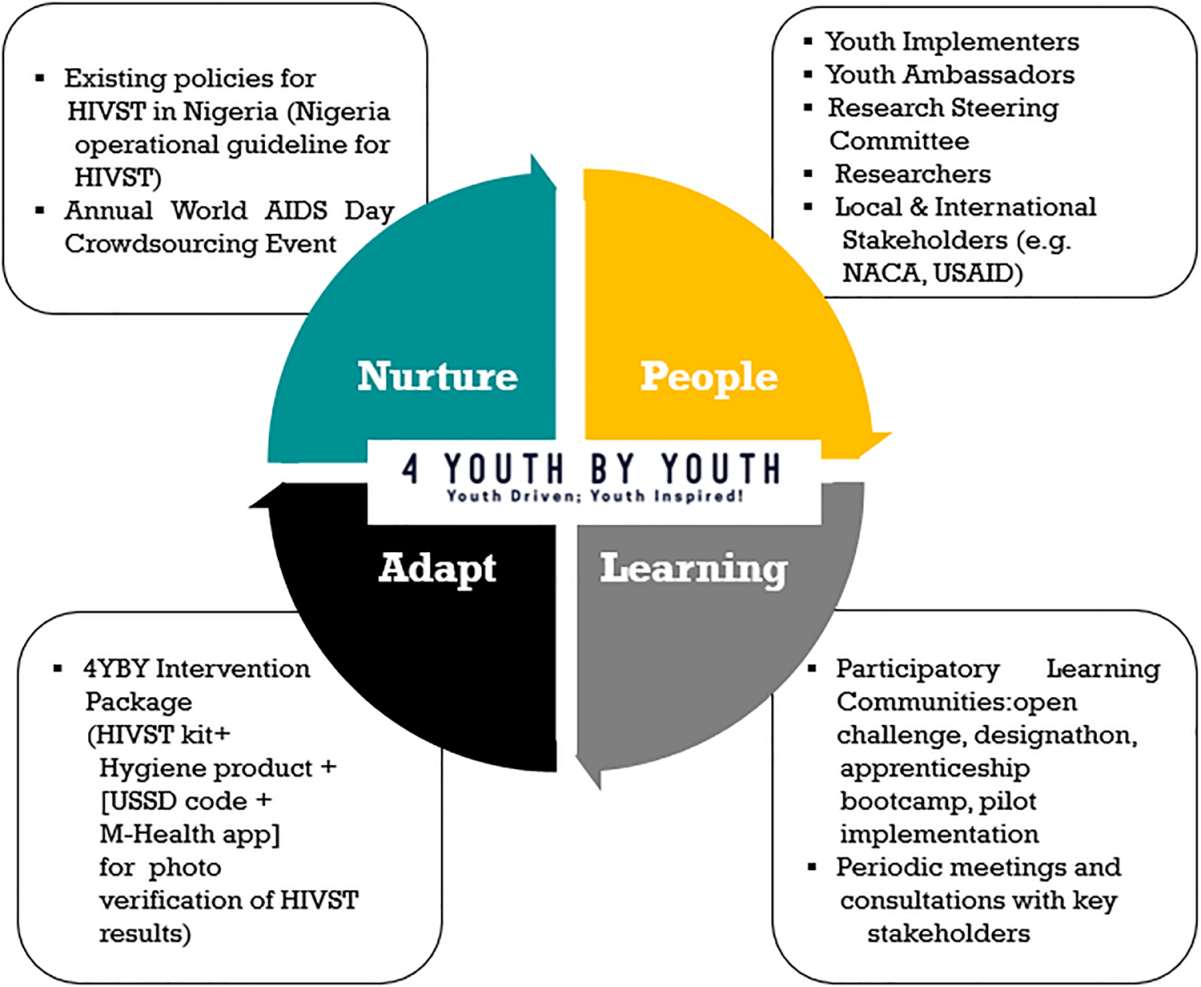
4YBY PLAN activities. Note: HIVST, HIV self-testing; NACA, National AIDS Control Agency (Nigeria); USAID, United States Agency for International Development; USSD, unstructured supplementary service data

The ongoing 4YBY project is a pragmatic trial seeking to address the HIV prevention gap among Nigerian youth [57]. Tapping into the wisdom of youth, we used implementation strategies such as participatory learning communities that combined youth-friendly open challenges, designathons, and apprenticeship bootcamps. They were utilized to design and pilot test developmentally and contextually appropriate strategies to promote the uptake and long-term sustainability of HIV self-testing among youth populations in Nigeria. Our participatory learning community is an adaptation of the learning collaborative (LC) implementation model, which brings teams from different organizations to work together to learn about an evidence-based intervention and sustain its use over time. We also extend LC in two important ways. First, many interventions targeting youth populations have generally focused on enhancing youth educational opportunities instead of training young people to develop innovative health services. Our participatory learning community is among the first to examine how youth can be prime movers in developing, implementing, and ultimately sustaining evidence-based interventions in a resource-limited setting. Second, the researcher’s innate tendency to groupthink even within learning collaboratives results in top-down solutions that are pushed out to youth populations assuming behavior change will occur regardless of fit. Our participatory learning communities use bottom-up, youth-friendly participatory approaches to engage youth and spur health solutions that have the greatest salience to youth themselves, thus creating an enabling environment to expand health services for youth. While the pragmatic trial is ongoing, here we introduce how PLAN has informed efforts to sustain 4YBY from the onset. An overview of the application of PLAN in the 4YBY project is presented in Fig. 2.

### Core Values

First, a core group of stakeholders, including a youth advisory board (i.e., youth ambassadors), helped to identify our core value as working with young people to help create interventions that are “youth-driven, youth-inspired.” Our core value was also informed by our use of youth participatory action research, which considers youth not only as beneficiaries or partners but as potential leaders and co-creators of knowledge [58, 59]. Our core value for the research project is also evident in the name “4 Youth By Youth.” 4YBY has youth as the inspiration and key driving force to identify and create health solutions that matter for themselves and their peers. Other values include the health benefits to young people themselves (i.e., increased uptake of HIV self-testing), capacity needed to continue aspects of intervention (i.e., annual World AIDS Day event), time (i.e., over 6 months to 24 months), and dissemination strategies (i.e., via social media, abstracts). These values direct the project activities, from the selection of key stakeholders to intervention design and implementation.

### People

Following the identification of the project’s core values, the next step was to identify and gather the key people to foster continued use of intervention components among young people. With the goal to sustain our youth-driven and youth-inspired HIV self-testing delivery strategies, we identified key stakeholders and potential program champions likely to be crucial using a stakeholder mapping analysis at the start of the project. Some of the key stakeholders included the Nigeria Federal Ministry of Health, local government area leaders, the Nigerian AIDS Control Agency, the Lagos State AIDS Control Agency, Community Alliances (i.e., Nigeria Youth Network on HIV/AIDS—a youth coalition to promote HIV prevention and management in Nigeria), and 4YBY youth ambassadors (a youth advisory board who provide input and feedback on all research activities to ensure that they are appropriate and acceptable for Nigerian youths), and Co-creation Hub for the development of the HIV self-testing mobile phone verification application. The identification and involvement of these stakeholders helped to foster ownership and fit of the youth-led HIV self-testing interventions to the local context. In addition to national and local agencies, we identified and engaged with several international organizations that work in Nigeria (i.e., UNAIDS, UNICEF, Google Nigeria) as key stakeholders of the research. Finally, and leveraging existing research resources in Nigeria, our local implementers are the Nigerian Institute of Medical Research, the apex research establishment in the country. NIMR is responsible for leading and coordinating all implementation, thus enhancing the opportunities for continuation of intervention components over time.

### Learning

In addition to identifying the key people, learning is at the core of 4YBY implementation in Nigeria. Learning is both a collaborative and iterative process, and for 4YBY, it occurred both at the individual participant level and the organization/setting level. At the individual participant level, we utilized learning communities as an implementation strategy whereby young people worked together to collectively solve problems on ways to expand HIV self-testing to their peers, learn best practices, apply quality improvement methods in the process of implementation, and exchange experiences with implementation [60]. Our learning communities were designed to be participatory, youth-friendly. It included open challenges such as crowdsourcing and designathon focused on generating ideas to expand HIV self-testing, as well as apprenticeship bootcamp, and pilot implementation focused on working with youth implementers to implement interventions they designed. For example, the open challenges involved Nigerian youths working as teams to develop strategies to promote HIV self-testing among young people in Nigeria. The apprenticeship bootcamp trained youth teams with the skills necessary to implement and evaluate their interventions among young people in real-time. In the end, five youth-led HIVST interventions were piloted tested in the community. Overall, our learning communities fostered a sense of ownership for and with young people to be actively engaged in activities to promote uptake of HIV testing among Nigerian youths. Likewise, at the setting level, we continue to foster opportunities for learning through periodic meetings with research staff and key stakeholders and booster sessions with research staff. Prior to starting our intervention implementation, we organized stakeholder consultations with key local stakeholders to ensure that the 4YBY activities aligned with the revised Nigerian HIV/AIDS Strategic Framework [61] and the PEPFAR Country Operational Plan [62]. In addition, and throughout the lifecycle of the intervention, we continually conduct booster sessions with research staff to ensure that aspects of the intervention are implemented as intended.

### Adapting

With adaptation and alignment with the National HIV Strategy for Adolescents and Young People [63], 4YBY worked with youth teams to co-create, refine, and pilot test a minimum package of HIV Prevention Services that targeted youth populations in Nigeria. It included HIV self-testing kits and linkage to youth-friendly health services for Sexually Transmitted Infection services. Additionally, 4YBY was originally conceived to collect data on self-reported uptake of HIV self-testing and STI testing among youth participants. But throughout implementation and during our learning communities with youth participants, our team identified the need to create a mobile application that would enable photo verification of HIVST results and linkage to youth-friendly prevention services for additional STI testing and treatment if needed. Utilizing FRAME-IS [46, 47] as a guide, our modification occurred during the pre-implementation phase of our project. Specifically, we collaborated with a local innovation hub in Nigeria, known as Cc-HUB, to plan the design and pilot-testing of a mobile photo-verification app that will be utilized during the pragmatic trial to report uptake of HIVST. The content of the mobile application was co-created with youth participants, and additional usability tests were conducted with them to ensure that the application was feasible to use, acceptable, and appropriate among youth participants. In addition, and to increase the reach of our intervention, another adaptation led by members of a youth team was the design and pilot-testing of a USSD (unstructured supplementary services data) code that will now be utilized during our pragmatic trial to assess uptake of HIVST and linkage to youth-friendly health services. A USSD code is a real-time communication technology used in sending messages across a Global System for Mobile Communications (GSM) network between a mobile client and an application server [64, 65]. Unlike SMS (short messaging services), USSD involves simple operations that are also handset independent (i.e., does not rely on smartphones), more secure than SMS, affordable, and interactive in nature [64, 65]. As part of their design and implementation strategy during the pilot implementation phase of the project, a youth implementer team developed the USSD codes to increase the reach of intervention among youth participants who may not have access to smartphones. Nonetheless, both adaptations, the mobile photo-verification app and the USSD, were inspired and driven by young people themselves. Together with local key stakeholders such as Cc-HUB, our planned adaptations to the pragmatic trial ensures community ownership and fit, revealed opportunities, and are consistent with the norms and interests of the youth population.

### Nurturing

With nurturing, 4YBY uses existing resources and supporting structures to spur continuous feedback via ongoing dialogue around its sustainability. Documents like the Nigerian National HIV/AIDS Strategic Framework and the National HIV Strategy for Adolescents and Young People guided the research activities, and engagement of key stakeholders. For example, following dialogue and engagement with key stakeholders such as NACA and LSACA, the goals and outcomes of 4YBY were aligned squarely with the existing policy documents such as the Nigerian National HIV/AIDS Strategic Framework [66]. Indeed prior to our arrival, the 2017 policy document made no reference to HIV self-testing [66]. But via advocacy led by our local principal investigator, the current 2019 policy document explicitly highlights the need to enhance demand and increase uptake of HIV testing among youth populations with recommendations on the use of HIVST as an additional approach for the delivery of HIV testing services to youth population [61]. 4YBY builds upon these existing resources as opportunities to apply continuous feedback, engage in midcourse strategic corrections, and continuous improvement that will enable us to generate demand and increase uptake of HIV self-testing services designed and implemented by young people themselves [23]. In addition, we tapped into existing activities led by NIMR and LSACA as part of the agencies ongoing World AIDS Day programming, to launch and implement an annual 4YBY HIV self-testing contest. Working with our partners, we use the opportunity to not only hear about scientific progress that agencies such as NIMR and LSACA have made to prevent the spread of HIV but what young people themselves can do on their own to develop solutions that lead to an AIDS-free generation. We realize that we will never be able to take full advantage of the scientific progress aimed at reducing HIV within Nigeria if the same efforts are not equally made to develop, nurture, and sustain youth voices and capacities necessary to implement these scientific breakthroughs, particularly in the area of HIV self-testing. NIMR and LSACA have now made joint commitments to maintain the 4YBY HIV self-testing contests annually until the vision of an AIDS-free generation is realized in Nigeria.

## Discussion

Bringing attention to sustainability as a core component embedded within an intervention overall cycle requires planning. We have demonstrated with case-study evidence from the 4YBY project that sustainability planning can be made simple with a dynamic plan that draws attention to assembling the key people while learning, adapting, and nurturing the core values of evidence-based intervention components or activities from the onset of implementation and overtime. Previous research indicates that the most crucial way to ensure sustainability is to be clear on the intervention’s values then establish goals and objectives needed to accomplish said values [23]. With values in place, we see PLAN as a guiding consideration for sustainability into all stages of intervention implementation, pre-implementation, implementation, and post-implementation. It also involves making decisions about how to deploy the values and is a process that typically gets revisited through the lifespan of an intervention [23]. A focus on values does not mean that all aspects of an intervention will continue. Only the core or key ingredients of an intervention, with attention to these elements throughout the intervention implementation can enhance sustainability. Additionally, active and early planning will allow sustainability to move from a latent goal, where researchers wish aspects of their interventions to continue, to one where conditions vis-à-vis the people, the learning, the adaption, and the nurturing that would most enhance prospects of sustainability long-term are considered whether with core elements of the intervention, continuation of intended benefits, and adequate capacity for continuation is maintained [5]. Conceptually, PLAN then might relate to sustained program → sustained benefits → sustained capacity → sustained value, but in the absence of early and active planning, none of this will occur [67].

Nevertheless, much work remains to be done to understand how PLAN contributes and advances the research agenda sustainability over longer periods of time. It will also require additional streams of data, particularly from implementation trials that specifically target sustainability as a process, incorporating concepts of learning, adaptation, and continuous improvement or nurturing of existing resources within particular contexts. If these implementation studies carefully think long and deep about the core values of evidence-based interventions, and rigorously plan from the onset to even wean off their projects to the key people, learning from them, adapting where necessary, and nurturing existing resources overtime, then the vision of the continued use of these components for the continued achievement of desirable health outcomes within a population of interest. We are closer than ever before to facilitating the how-to-do-it literature on the sustainability process of evidence-based interventions, but it will require a theory-informed and dynamic PLAN, at the bare minimum. Thus, we call for future application across different populations and contexts, and mixed-methods research to evaluate and further refine the PLAN approach for sustainability planning throughout the life cycle of an EBI.

## Data Availability

Not applicable.

## Abbreviations

*4YBY*: 4 Youth By Youth
*Cc-HUB*: Co-creation Hub
*DSF*: Dynamic Sustainability Framework
*FRAME*: Framework for Reporting Adaptations and Modifications Enhanced
*GSM*: Global System for Mobile Communications
*LC*: Learning collaborative
*LSACA*: Lagos State AIDS Control Agency
*NACA*: Nigerian AIDS Control Agency
*NIMR*: Nigerian Institute of Medical Research
*NYNETHA*: Nigeria Youth Network on HIV/AIDS
*PLAN*: How People can Learn to Adapt and Nurture the core values of evidence-based interventions overtime
*RE-AIM*: Reach, Effectiveness, Adoption, Implementation, and Maintenance
*SMS*: Short messaging services
*USSD*: Unstructured supplementary services data
